# The role of Sirtuin 1 in the pathophysiology of polycystic ovary syndrome

**DOI:** 10.1186/s40001-022-00746-4

**Published:** 2022-08-27

**Authors:** Mali Wu, Jie Zhang, Ran Gu, Fangfang Dai, Dongyong Yang, Yajing Zheng, Wei Tan, Yifan Jia, Bingshu Li, Yanxiang Cheng

**Affiliations:** 1 Department of Obstetrics and Gynecology, Renmin Hospital of Wuhan University, Wuhan, 430060 Hubei China; 2 Department of Pain, Renmin Hospital of Wuhan University, Wuhan, 430060 Hubei China

**Keywords:** PCOS, SIRT1, Oxidative stress, Autophagy, Treatment

## Abstract

Polycystic ovarian syndrome (PCOS) is the most common multifactor heterogeneous endocrine and metabolic disease in women of childbearing age. PCOS is a group of clinical syndromes characterized by reproductive disorders, metabolic disorders, and mental health problems that seriously impact the physical and mental health of patients. At present, new studies suggest that human evolution leads to the body changes and the surrounding environment mismatch adaptation, but the understanding of the disease is still insufficient, the pathogenesis is still unclear. Sirtuin 1 (SIRT1), a member of the Sirtuin family, is expressed in various cells and plays a crucial role in cell energy conversion and physiological metabolism. Pathophysiological processes such as cell proliferation and apoptosis, autophagy, metabolism, inflammation, antioxidant stress and insulin resistance play a crucial role. Moreover, SIRT1 participates in the pathophysiological processes of oxidative stress, autophagy, ovulation disturbance and insulin resistance, which may be a vital link in the occurrence of PCOS. Hence, the study of the role of SIRT1 in the pathogenesis of PCOS and related complications will contribute to a more thorough understanding of the pathogenesis of PCOS and supply a basis for the treatment of patients.

## Introduction

**Fig. 1:**
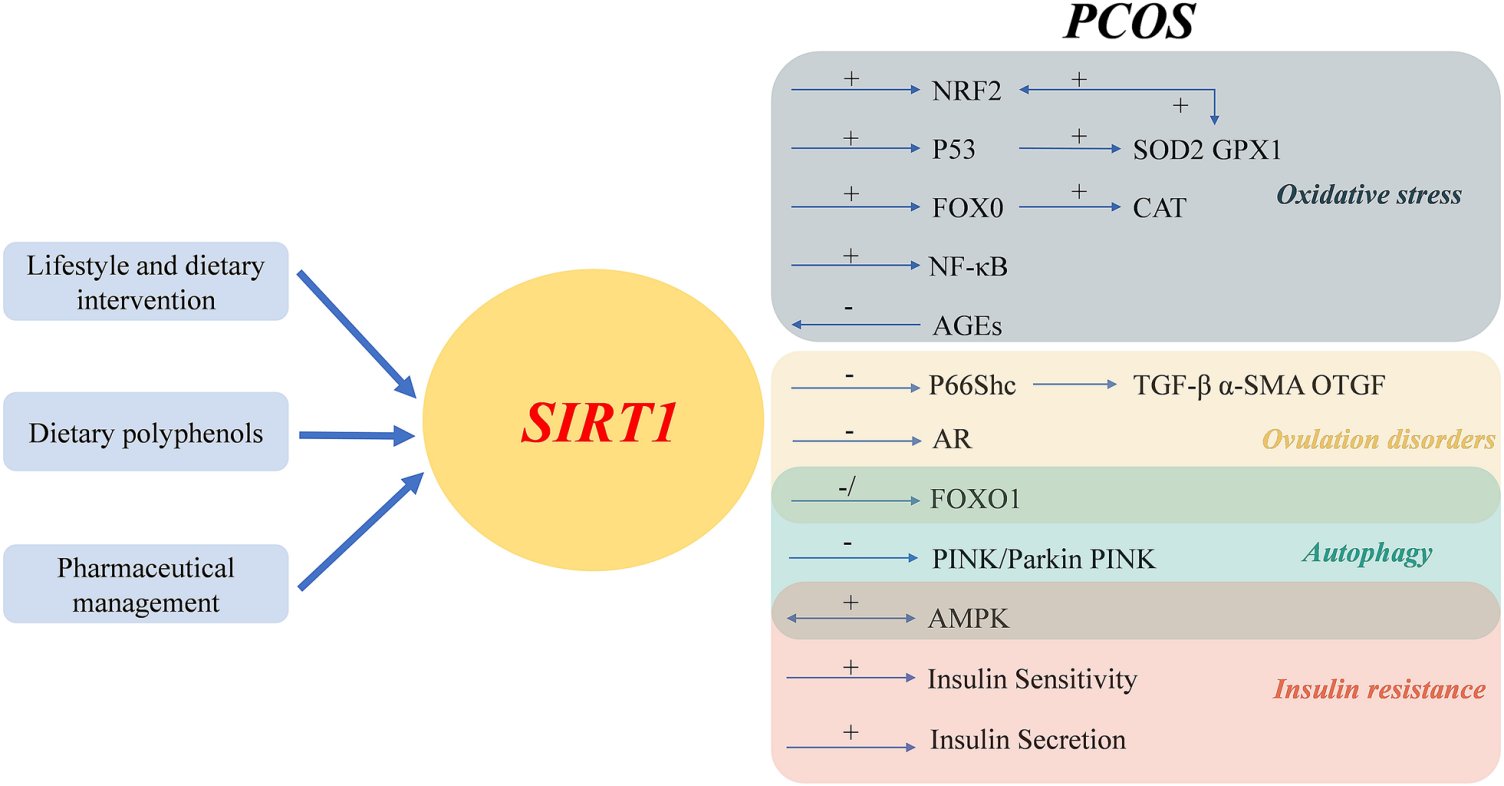
Role of SIRT1 in the pathophysiology of polycystic ovary syndrome

Polycystic ovary syndrome (PCOS) is an endocrine and metabolic disease characterized by hyperandrogenemia, ovulation disorder and ovarian polycystic transformation. Approximately 10–20% of women worldwide suffer from this disease, affecting their quality of life [1–3]. Patients with PCOS showed chronic inflammation and oxidative stress. Its metabolic damage is complex, including insulin resistance (IR) and compensatory hyperinsulinemia, which have major effects on muscle and adipose tissue, and are closely connected with other metabolic diseases such as inherent β-cell dysfunction, type 2 diabetes mellitus (T2DM), gestational diabetes mellitus, increased risk factors for cardiovascular disease (hypertension, hyperlipidemia, etc.), obesity and metabolic syndrome (METS) [4–7]. Nevertheless, existing studies ignore the growing acceptance of evolutionary perspectives, the role of lifestyle and diet, the role of androgens in the origin of PCOS development, the influence of the microbiome, and the reversibility (Fig. 1) of metabolic, biochemical, and endocrine factors of PCOS following lifestyle and other interventions. To some extent, the diagnosis and treatment of the disease are limited, and the prevention and treatment effect is not good.

Sirtuins, a family of nicotinamide adenine dinucleotide + (NAD +)-dependent deacetylases, are the key metabolic receptors of homeostasis in the human body [8]. Silencing information regulator 2-related denzyme 1 (Sirtuin 1, SIRT1) can regulate cell metabolism, senescence, and antioxidant stress by deacetylating transcription factors, coregulatory factors, and histones, to inhibit cell apoptosis, oxidative stress, and inflammation [9–11]. The decrease in SIRT1 activity or the inhibition of its related pathway is a common pathological process in nonalcoholic fatty liver, cardiovascular disease, and other metabolic and inflammatory diseases. Previous studies have shown that activating SIRT1 can benefit the treatment of many diseases as a new target [8].

Are SIRT1 and related pathway molecules involved in the pathogenesis of PCOS? What is the specific role of SIRT1 in endocrine, reproductive and metabolic disorders in patients with PCOS? Can the symptoms of PCOS patients be improved by regulating SIRT1 and its pathway molecules? In recent years, extensive research has been devoted to exploring the function of SIRT1 in PCOS, providing new inspiration for the treatment of disease in the future, and offering a scientific basis for clinical application.

## Structure and function of SIRT1

Sirtuins 1–7 are widespread and conserved class of NAD + -dependent histone deacetylases in mammals [12]. According to different subcellular localizations, they can be divided into four categories: SIRT1, SIRT2 and SIRT3 belong to class I, SIRT4 belongs to class II, SIRT5 belongs to class III, and SIRT6 and SIRT7 belong to class IV, which can act on different substrates [13]. They can be involved in cell proliferation, metabolism, transcription, apoptosis and cell signal transduction [14, 15].

Human SIRT1 expressed in the nucleus and encoded by the SIRT1 gene is located on chromosome 10q21.3, functioning in the deacetylation of the histone and nonhistone lysine groups of known transcription factors (FOXO, MyoD, p53, PGC-1a) [16]. SIRT1 connects transcriptional regulation with intracellular energetics, coordinates different cellular functions, and goes far beyond simple histone deacetylation [17]. The dysfunction will bring about tissue-specific degenerative changes, which are the pathological basis of many diseases, including cancer, cardiovascular disease, type 2 diabetes, and many other diseases [18, 19]. SIRT1-mediated deacetylation activates liver kinase B1 (LKB1) signals in the cytoplasm and can further add fatty acid oxidation in the liver [20]. SIRT1 is also involved in the balance of cholesterol metabolism in the liver. The process balance disorder may lead to intrahepatic fat accumulation [19–21]. SIRT1 can mediate the expression of tumor-related genes, such as apoptosis protein inhibitor (IAP), through nuclear factor kappa B (NF-kB) to participate in tumorigenesis [22]. SIRT1 can modulate mitochondrial function, glucose metabolism, and lipids by activating peroxisome proliferator-activated receptor-gamma coactivator (PGC-1a) gene transcription and regulating peroxisome proliferator-activated receptor (PPAR), nuclear respiratory factor (NRF) and mitochondrial transcription Factor A (TFAM), which are closely related to the occurrence of metabolic syndromes, such as insulin resistance [23].

## The relationship of SIRT1 and PCOS 

The occurrence of PCOS involves in multiple pathways and lacks common clues, causing different symptoms in patients. Patients with PCOS may be in a state of low-grade inflammation and oxidative stress, often accompanied by clinical manifestations of endocrine, reproductive and metabolic disorders, such as menstrual disorder, hyperandrogenemia, infertility, obesity, insulin resistance, ovarian changes, hirsutism, acne and more [24, 25]. However, the existing evidence shows that there is a significant relationship between PCOS and Sirtuin 1 genetic polymorphism [26]. For the past few years, emerging studies have focused on the role of SIRT1 in the pathophysiological process of PCOS.

### SIRT1 and oxidative stress

Oxidative stress is the imbalance between oxidants and antioxidants and the production of excessive reactive oxygen species (ROS) [27]. An increasing number of studies have shown that active oxygen will be overproduced, the level of biomarkers of circulating oxidative stress will increase, and the antioxidant capacity will gradually decrease in patients with PCOS [28–30]. In addition, PCOS patients also have mitochondrial dysfunction, Redox potential imbalance and increased oxidative stress levels are observed in cumulus cells [31]. Currently, SIRT1 has been found to be able to protect against PCOS by reducing the expression of oxidative stress markers and methylglyoxal (MG), which is closely related to glycosylation stress, and improving mitochondrial disorders [32].

P53, forkhead box O (FOXO) and nuclear factor NF-kappa B (NF-κB) are the core targets of SIRT1-mediated redox state alteration [33]. P53, a transcription factor, can activate antioxidant defense-related genes, such as superoxide dismutase 2 (SOD2) and glutathione peroxidase (GPX1). FOX03a induces an antioxidant response by upregulating catalase [34, 35]. SIRT1 can also stabilize antioxidation by upregulating nuclear factor erythroid 2 (NRF2) by deacetylating nuclear Factor E2-related Factor 2 and promoting the expression of SOD, catalase (CAT) and glutathione (GSH) [36]. Advanced glycation end-products (AGEs) have been shown to bind to the multiligand receptor for advanced glycation end products (RAGE) to activate important intracellular signaling pathways and induce the production of oxidative stress-related factors and proinflammatory cytokines [37]. Increasing ROS levels and the inflammatory response aggravate endocrine and metabolic disorders in PCOS [38]. In a PCOS mouse model, MG accumulation can lead to the imbalance of SIRT1, decreasing the expression of protective factors related to mitochondria (PGC1 α, MtTFA, TOMM20) [39]. In contrast, the balance of SIRT1 was confirmed to have a protective effect on mitochondria and further protect cells from oxidative stress.

### SIRT1 and autophagy

Autophagy is a type of cell death recently identified in PCOS ovarian cells, characterized by the phagocytosis of cytoplasmic material into two-membranous vesicles (autophagosomes) and subsequent degradation in lysosomes [40]. Excessive autophagy is the self-destruction of cells when they are subjected to oxidative damage, which can be manifested by mitochondrial dysfunction or structural changes [41, 42]. The degree of autophagy in PCOS patients, rat ovarian tissue and PCOS cell model was significantly increased. For example, Chuyue Zhang et al. found that high migration framework 1(HMGB1) can induce increased autophagy in granulosa cells of PCOS patients, thus aggravating insulin resistance [43]. Other indicators of significant change are mitochondrial membrane potential, mtDNA content, and decreased protein level of the autophagy substrate p62; however, the number of autophagosomes and the levels of the autophagy markers Beclin1 and LC3B-II increased [44, 45].

SIRT1 can regulate the deacetylation of LC3, an important autophagy mediator, suggesting that SIRT1 plays an important role in the regulation of autophagy [46]. Previous studies have also demonstrated that SIRT1–FOXO1 plays a critical role in the regulation of autophagy [47]. Giovanna Di Emidio et al. found that SIRT1 expression and adenosine monophosphate-activated protein kinase (AMPK) activation were significantly enhanced in the ovary in the established dehydroepiandrosterone (DHEA)-induced PCOS mouse model, suggesting that SIRT1 may regulate PCOS ovarian autophagy through activation of AMPK [48]. In addition, activation of SIRT1 inhibits PTEN-induced putative kinase 1 in granulosa cells (GCs) of PCOS patients, thereby protecting mitochondria from damage, reducing the level of ovarian autophagy, and improving oxidative stress [48]. In conclusion, when activated by external factors, SIRT1 can prevent autophagy and mitochondrial damage by inhibiting autophagy-related molecules, thus promoting the body's antioxidant effect and protecting mitochondria and cells from the adverse effects of oxidative stress.

### SIRT1 and ovulation disorders

Ovulation disorders account for approximately 30% of infertility, and are usually characterized by irregular menstruation (less menstruation) or no menstruation (amenorrhea) [49]. In the reproductive system, Xian Qin et al. found that ovarian reserve was positively correlated with an increase in SIRT1 expression in mice, suggesting that SIRT1 can delay ovarian aging [50]. Other experiments have proven that SIRT1 can inhibit FOXO1 acetylation to promote the decomposition of the Fox01–ATG7 complex, reduce the autophagic death of GCs under oxidative stimulation, and delay the senescence of oocytes [51, 52]. Likewise, in a rat model, SIRT1 activation can not only suppress the expression of androgen receptor and decrease the level of androgen but also keep down p66Shc expression, thus maintaining TGF-β, α-SMA and CTGF expression and reforming the structural fibrosis of the ovary [53]. On all accounts, SIRT1 has great potential in ameliorating ovulation disorders in PCOS, and more in-depth research on its mechanism is needed.

### SIRT1 and insulin resistance (IR)

Worldwide, 1 in 6 to 20 women of reproductive age (5 to 20%) who exhibit hyperandrogenemia in PCOS are affected by insulin resistance (IR) or hyperinsulinemia [54]. IR is a pathological metabolic state in which the ability of the body to use glucose decreases to compensate and maintain normal blood sugar levels and increase insulin secretion, resulting in hyperinsulinemia. SIRT1 positively regulates insulin secretion in pancreatic β-cells [55]. Moreover, increased expression of SIRT1 improved insulin sensitivity, especially under insulin-resistant conditions [56].

Studies have shown that the levels of AMPK (the key regulator of the mitochondrial response to energy deprivation) and SIRT1 in the ovaries of PCOS rats are significantly lower than the levels of AMPK of the control group, and they are in an obvious IR state, which is the same as in PCOS mice [57–59]. However, when the expression of AMPK and SIRT1 is significantly increased, it can reduce blood sugar and protect microvascular endothelial cells from glucose toxicity [57–60]**.** In other studies, a potent small molecule activator of SIRT1 reduced blood glucose and improved insulin sensitivity in mice with diet-induced obesity [61]. AMPK and Sirtuins are present in all eukaryotic cells and may have coexisted during evolution [62, 63]. AMPK enhances SIRT1 expression by regulating nicotinamide activity, and SIRT1 also activates AMPK [64, 65]. In conclusion, the AMPK–SIRT1 pathway may be the molecular mechanism of IR in PCOS and may serve as a therapeutic target for developing potential therapies to improve the metabolism and reproductive function of PCOS.

## Overview of the role of SIRT1 in the treatment of PCOS

Multiple lines of evidence now suggest that in the modern world, there are maladaptive reactions in humans to rapidly changing nutritional, physiological, psychological and cultural environments, which lead to pathological responses to IR, hyperandrogens, enhanced energy storage and ovulation [66, 67]. SIRT1 is conserved throughout evolutionary history, as a cellular metabolic energy sensor, right back to the beginning of eukaryotic organisms. Sirtuins constitute a family of metabolic sensor proteins that translate changes in NAD+levels into adaptive responses and play an important regulatory role in lipid glucose metabolism and mitochondrial activity [68]. SIRT1 is an NAD-dependent histone deacetylase that is activated when there are low cellular energy levels that result in an elevated NAD + to NADH ratio, which occurs between meals and during fasting and leads to the activation of multiple catabolic pathways, inhibition of anabolic pathways (with activation of AMP kinase and inhibition of mTOR), and activation of cellular processes, such as autophagy (as discussed in Sect. 3.2). Previous research established that the treatment strategy of activating SIRT1 can be applied to the treatment and life management of patients with PCOS to further improve symptoms. To date, the application of SIRT1 in PCOS treatment is in the exploratory stage, which basically includes the following aspects: 1. Lifestyle and dietary intervention; 2. Supplement of dietary polyphenols; and 3. Pharmaceutical management.

### Lifestyle and dietary intervention in the management of PCOS

The activation of SIRT1 and the subsequent cellular changes are, therefore, dependent on nutritional energy intake and activity levels, which highlights the central role of lifestyle factors, such as diet and exercise in the pathogenesis of PCOS (as elaborated on in the 2018 International Guidelines) [69]. Data from several studies suggest that regular exercise and a whole food diet can regulate SIRT1 activity and have effects on weight loss and metabolic and clinical biomarkers [70]. Appropriate sun exposure can promote the synthesis of vitamin D, improve metabolic parameters, and increase SIRT1 activity [71, 72]. A number of other ways of activating SIRT1 by changing diet and lifestyle have been investigated, including increased intake of docosahexaenoic acid (DHA), polyphenols, extra virgin olive oil, and moderate cold stimulation [73–76]. Some of these factors are potentially important components of a healthy lifestyle and need further clinical investigation.

### The role of dietary polyphenols in the management of PCOS

Polyphenols undergo intensive biotransformation by the gastrointestinal microbiota, and less than 5% of ingested polyphenols are estimated to reach the circulation intact [77]. A large number of microbial polyphenol metabolites can be detected in plasma compared with extremely low levels of the parent compounds. Despite their low bioavailability, numerous studies have reported significant biological effects related to dietary polyphenols in women with PCOS. These effects include resveratrol, quercetin and curcumin. The anti-inflammatory and oxidative stress effects of polyphenols can effectively reduce the incidence of chronic diseases, such as obesity, diabetes and cardiovascular diseases in the population. Supplementing natural compounds through diet or other means can effectively reduce the adverse effects of related diseases [19]. A large number of experiments have proven that bioactive substances such as polyphenols can play a protective role by regulating SIRT1 expression and activity in vivo, which is a potential way to treat or prevent metabolism-related diseases [78, 79]. The activation of SIRT1 to improve symptoms in PCOS patients not only provides a new target for treatment but also further validates the pathogenesis of PCOS.

#### Resveratrol

Resveratrol can remove ROS, inhibit cyclooxygenase (COX), and activate anti-inflammatory and antioxidant stress pathways through SIRT1 [80]. In a rat control experiment, resveratrol (20 mg/kg/d) decreased body weight and ovarian weight, reduced the levels of testosterone, luteinizing hormone (LH), LH/follicle stimulating hormone (FSH), tumor necrosis factor (TNF)-α and tissue antiMüllerian hormone (AMH), and affected the maintenance of follicular formation [81]. Another set of rat models of PCOS induced by high androgens (dehydroepiandrosterone and dihydrotestosterone) found that resveratrol significantly reduced ovarian oxidative stress levels, inhibited phosphorylation of p66Shc, inhibited fibrotic factor activation, and improved ovarian morphology [53].In clinical trials of women with PCOS, resveratrol has been shown to improve ovarian volume, high-quality oocyte rate, high-quality embryo rate, androgen and gonadotropin concentrations, angiogenic factor levels, and endoplasmic reticulum stress levels in PCOS patients [82].In two randomized controlled trials of patients with nonalcoholic fatty liver disease and obesity, resveratrol combined with a low-calorie diet or exercise significantly reduced body weight and improved serum levels of total cholesterol (TC), high density lipoprotein cholesterol (HDL-C), very low density lipoprotein cholesterol (VLDL-C), urea, creatinine, and albumin compared with diet control or exercise alone. PCOS is suggested to be able to be used in weight management and treatment of metabolic disorders [83, 84].

#### Quercetin

Quercetin may exert anti-inflammatory, antiapoptotic, antioxidant and anticancer effects mainly through the SIRT1/AMPK axis and can enhance oocyte and embryo quality in the ovary [85, 86]. For the past few years, tests on the therapeutic effects of quercetin on PCOS and ovarian cancer have been carried out. In a letrozole-induced rat PCOS model, the expression of AMPK and RT-1 in ovarian tissue was upregulated in the quercetin (100 mg/kg) treatment group, and the PCOS-related estrus cycle, lipid profile, serum testosterone, estradiol and progesterone levels, and IR disorders were improved. The changes in adiponectin, adiponectin and resistin in adipose tissue induced by PCOS were also reversed to a certain extent [85]. After quercetin treatment, the following changes occurred in the rats with PCOS induced by dihydrotestosterone: the activity of progesterone, metabolic enzymes and antioxidant enzymes was significantly increased, and DHEA-induced morphological changes related to polycystic ovaries were alleviated [87]. Quercetin also significantly decreased the expression of testosterone (T), estradiol (E(2)), LH, Bax, IL-1β, IL-6 and TNF-α, increased the expression of FSH and Bcl-2, and inhibited the expression of AR. By affecting the binding of androgen receptor (AR) to specific sequences of (C-type natriuretic peptide) CNP and (natriuretic peptide receptor 2) NPR2 gene promotors, the expression of CNP/NPR2 genes and proteins is regulated to restore oocyte maturation and ovulation [88]. Controlled trials in overweight or obese PCOS patients have shown that quercetin can significantly reduce serum testosterone, luteinizing hormone, and serum inhibin levels and expression and improve insulin resistance [89]. Quercetin can also enhance the expression of adiponectin receptor transcripts in PCOS patients and effectively improve adiponectin-mediated insulin resistance and hormone metabolism disorder [90, 91].

#### Curcumin

Other substances that enhance SIRT1 activity include curcumin [19], which is one of the main polyphenol compounds in turmeric, with antioxidant, anti-inflammatory, anticancer, antiarthritis, antiasthma, antimicrobial, antiviral and antifungal properties and has potential benefits for the treatment of female reproductive diseases [92].Previous studies focused on the therapeutic potential and mechanism of curcumin on PCOS by constructing curcumin nanoparticles: The use of curcumin (Cur) coated with arginine (Arg) and N-acetylhistidine (Nache)-modified chitosan (ARG-Cs-Nache/Cur) nanoparticles (NPs) in estradiol valerate (EV)-induced PCOS rats reversed multiple symptoms of PCOS [93]. Curcumin nanocapsules can improve insulin resistance and lipid profiles in conjunction with metformin in PCOS patients [94]. Other related studies have also shown that curcumin can improve the metabolic disorders of PCOS patients, which is beneficial to their weight control and reduces serum inflammatory markers [95, 96] and may be a safe and effective supplement for improving PCOS-associated hyperandrogenemia and hyperglycemia [97].

Current studies indeed show that the antioxidant and anti-inflammatory effects of dietary polyphenols can be applied to the treatment of metabolic and inflammatory diseases, such as PCOS, and most of the substances can be ingested through food, which further demonstrates the important role of diet in disease management [19]. However, the mechanism of action of dietary polyphenols is still not thoroughly studied: whether it plays a role mainly through the activation of the SIRT1 pathway, the dose/dosage form required for its application in disease treatment and auxiliary programs, and safety still needs much clinical trial data.

### Pharmaceutical management of PCOS

Metformin can significantly improve insulin resistance and contribute to weight loss in PCOS patients [98]. In some studies, the mechanism of metformin alone or in combination with bioactive substances and other drugs to improve symptoms of patients has been further clarified: In rat experiments, it was found that the SIRT1 and AMPK immune reactivity were significantly increased and showed an increasing trend after metformin alone or in combination with resveratrol and exenatide, and the ovarian morphology and related metabolic indicators were significantly improved in PCOS rats. These results suggest that activation of SIRT1 may be an important pathway for metformin, exenatide, tayin-35 and other drugs and bioactive substances to treat PCOS patients [57–100]. However, the regulatory pathways and molecular mechanisms of SIRT1 activation by these pathways have not been fully studied. In the absence of a bridge between SIRT1 and the activator, increased SIRT1 activity can only be determined by downstream signals. Exploring the direct-action target of SIRT1 by molecular biology or histology research, and the development of high bioavailability, high specificity and clear SIRT1 target activator is the direction of future research.

In addition to the more in-depth study of the above drugs, new therapeutic drugs are being developed. Examples include glucagon-like peptide-1 receptor (GLP-1) agonists and sodium–glucose cotransporter 2 (SGLT-2) inhibitors [101]. Liraglupeptide (Lira) is a glucagon-like peptide-1 receptor agonist (GLP-1) that improves insulin sensitivity, reduces the risk of cardiovascular disease (CVD), leads to weight loss, and improves nonalcoholic fatty liver disease [102–104]. Lira has been shown to induce the expression of adenosine monophosphate activated protein kinase-α (AMPK-α) and SIRT-1 proteins and promote brown adipocyte differentiation and anti-inflammatory effects, thereby improving insulin sensitivity, reducing inflammation, and inducing adaptive thermogenesis [105]. In addition, sodium–glucose cotransporter 2 (SGLT-2) inhibitors, such as licogliflozin increase insulin sensitivity and ameliorate hyperinsulinemia and hyperandrogenemia in women with PCOS [106–108]. Unfortunately, there is currently a lack of studies on the interaction between SGLT2 inhibitors and SIRT1, which is also a new direction for future research.

## Conclusions

As a key hub of steady-state cellular energy metabolism in the human body, SIRT1 is not only related to the occurrence of cardiovascular and cerebrovascular diseases, such as fatty liver, but also is closely related to the occurrence and development of PCOS. In addition to the current relatively recognized pathogenesis of PCOS, oxidative stress/autophagy/hyperandrogenia/insulin resistance, this paper also considers the correlation between the generation of PCOS and the ancient evolutionary theory to further explore new views on the diet and lifestyle of the modern world and new treatment methods.

SIRT1 may protect PCOS patients, mainly through oxidative stress, inhibition of granular cell autophagy, improvement of mitochondrial dysfunction, abnormal improvement of ovulation disorders (enhanced quality of oocyte and embryo), improvement of the hormone metabolism disorder (lower testosterone levels), and a certain degree in improvement of its complications: obesity and lipid metabolic disorder. However, the current research direction of SIRT1 and PCOS exists only in the aspects of antioxidant stress, ovulation disorders, autophagy abnormalities, insulin resistance, etc., and many aspects remain to be explored. In addition, the current research level is relatively superficial, and it is only speculative based on the experimental results of existing studies, such as the clear treatment mechanism of SIRT1 and AMPK pathways in insulin resistance. The relationship between the SIRT1 expression level and the nutritional status of the human body and cells, the interaction mechanism between SIRT1 and new drug therapy, and the balance mechanism of its inhibition and promotion of autophagy need to be further studied and determined.

SIRT1-related PCOS treatment strategies are mainly to promote the activity of SIRT1 to exert the protective effects of antioxidant stress and anti-inflammatory pathways on PCOS patients. Specific plans include adjustment of diet and living habits, rational intake of bioactive substances and the use of drugs. However, the specific mechanism of enhancing SIRT1 activity in each scheme is still unclear, and there is a lack of clear molecular connection between dietary polyphenols and SIRT1 activity.

In the future, bioinformatics tools can be used to predict and verify molecular interactions and improve the drug action network. Specific dosage forms/dosages of dietary polyphenols in treatment regimens still need to include extensive data from animal and clinical trials, such as whether there is a difference in efficacy when nanotechnology is applied to drugs, such as quercetin. If natural compounds are used as treatment options, there is a lack of long-term observation and research on long-term patients' pregnancy and fetal safety. Further exploration of treatment plans will improve the understanding of SIRT1 and PCOS diseases, for example, the correlation between melatonin and biological rhythm, the correlation between circadian rhythm and PCOS, and whether these correlations affect autophagy in PCOS patients and further affect the disease phenotype. A fuller and more comprehensive understanding can help us search for SIRT1 modulators with high bioavailability and specificity and provide new efficient targets for the treatment and management of endocrine and metabolic diseases, such as PCOS.

## Data Availability

Not applicable.
